# *Leishmania major* Promastigotes Evade LC3-Associated Phagocytosis through the Action of GP63

**DOI:** 10.1371/journal.ppat.1005690

**Published:** 2016-06-09

**Authors:** Christine Matte, Pierre-André Casgrain, Olivier Séguin, Neda Moradin, Wan Jin Hong, Albert Descoteaux

**Affiliations:** 1 INRS-Institut Armand-Frappier and Centre for host-parasite interactions, Laval, Quebec, Canada; 2 School of Pharmaceutical Sciences, Xiamen University, Xiamen, Fujian, People’s Republic of China; 3 Institute of Molecular Cell Biology, Singapore, Singapore; Imperial College London, UNITED KINGDOM

## Abstract

The protozoan *Leishmania* parasitizes macrophages and evades the microbicidal consequences of phagocytosis through the inhibition of phagolysosome biogenesis. In this study, we investigated the impact of this parasite on LC3-associated phagocytosis, a non-canonical autophagic process that enhances phagosome maturation and functions. We show that whereas internalization of *L*. *major* promastigotes by macrophages promoted LC3 lipidation, recruitment of LC3 to phagosomes was inhibited through the action of the parasite surface metalloprotease GP63. Reactive oxygen species generated by the NOX2 NADPH oxidase are necessary for LC3-associated phagocytosis. We found that *L*. *major* promastigotes prevented, in a GP63-dependent manner, the recruitment of NOX2 to phagosomes through a mechanism that does not involve NOX2 cleavage. Moreover, we found that the SNARE protein VAMP8, which regulates phagosomal assembly of the NADPH oxidase NOX2, was down-modulated by GP63. In the absence of VAMP8, recruitment of LC3 to phagosomes containing GP63-deficient parasites was inhibited, indicating that VAMP8 is involved in the phagosomal recruitment of LC3. These findings reveal a role for VAMP8 in LC3-associated phagocytosis and highlight a novel mechanism exploited by *L*. *major* promastigotes to interfere with the host antimicrobial machinery.

## Author Summary

The early events surrounding and following the phagocytosis of pathogens largely determine whether internalization will lead to efficient killing of the microbe or successful establishment of an intracellular infection. Growing evidence supports the notion that the autophagy machinery lends a hand to phagocytosis in eliminating intracellular pathogens, in a process known as LC3-associated phagocytosis (LAP). Protozoan parasites of the *Leishmania* genus use surface virulence factors such as lipophosphoglycan and the metalloprotease GP63 to interfere with phagolysosome biogenesis and sabotage macrophage antimicrobial functions. Here, we provide the first evidence that *L*. *major* promastigotes evade LAP in a GP63-dependent manner and uncover a novel role for the membrane fusion mediator VAMP8 in LAP. Our findings offer a better understanding of *Leishmania* pathogenesis and of the mechanism behind LAP.

## Introduction

Phagocytosis plays a central role in linking innate and adaptive immunity [1, 2]. During this process, pathogens are internalized in a vacuole, the phagosome, which engages in a maturation program involving sequential interactions with various cellular compartments [1]. These interactions lead to the acidification of the phagosome and the acquisition of an array of hydrolases, culminating in the generation of a microbicidal phagolysosome. Peptides generated through the degradation of microbial antigens are processed in the phagolysosome, loaded on MHC molecules, and transported to the cell surface to initiate an adaptive immune response [1, 3, 4]. Phagolysosome biogenesis thus represents an important means of controlling infections, but several pathogenic microorganisms have evolved mechanisms to subvert this process and cause disease [5].

Upon their internalization by host phagocytes, promastigote forms of the protozoan parasite *Leishmania* turn off key antimicrobial and immune functions through the inhibition of phagolysosome biogenesis [6, 7]. The promastigote surface glycolipid lipophosphoglycan (LPG) plays a major role in this inhibition by disrupting phagosomal lipid microdomains [8–10]. Recent evidence indicates that the glycophosphatidylinositol (GPI)-anchored zinc-dependent metalloprotease GP63 also contributes to the *Leishmania-*induced phagosomal remodeling [7, 11]. Hence, during phagocytosis, GP63 is released from the parasite and rapidly gains access to various intracellular compartments where it cleaves a number of regulators of macrophage function [7, 11–16], including regulators of membrane fusion [7, 15]. One such molecule is the soluble N-ethylmaleimide-sensitive factor-attachment protein receptor (SNARE) Vesicle-associated membrane protein 8 (VAMP8), which we recently showed to control the early recruitment of the NADPH oxidase (NOX2) to phagosomes [7]. Targeting of VAMP8 through GP63 enables *Leishmania* promastigotes to inhibit assembly of the NOX2 complex on phagosomes, thereby impairing the ability of infected cells to process antigens for cross-presentation and to activate T cells [7, 17].

A non-canonical autophagic pathway termed LC3-associated phagocytosis (LAP) was recently shown to enhance the antimicrobial activity of macrophages by linking the autophagy pathway to phagocytosis [18–22]. This process, which is characterized by the recruitment of the autophagy-related protein LC3 to phagosomes, occurs during the internalization of particles that engage various receptors, including TLRs, FcR, TIM4, and Dectin-1 [18, 23–25]. Although the impact of LC3 recruitment on phagosomal functions remains to be fully elucidated, previous studies revealed that LAP enhances phagosome maturation and microbial killing [18, 23], as well as MHC class II-mediated antigen presentation [25]. One of the hallmarks of LAP is the requirement for reactive oxygen species (ROS) generated by NOX2 for LC3 recruitment to phagosomes [23]. Given the ability of *Leishmania* to interfere with assembly of the NOX2 complex during phagocytosis [7, 26], we sought to investigate the impact of *Leishmania* infection on LAP. Here, we provide evidence that *Leishmania* promastigotes evade LAP through a mechanism involving GP63-mediated cleavage of VAMP8 and exclusion of NOX2 from phagosomes.

## Results

### *Leishmania major* promastigotes impair the recruitment of LC3 to phagosomes in a GP63-dependent manner

**Fig 1:**
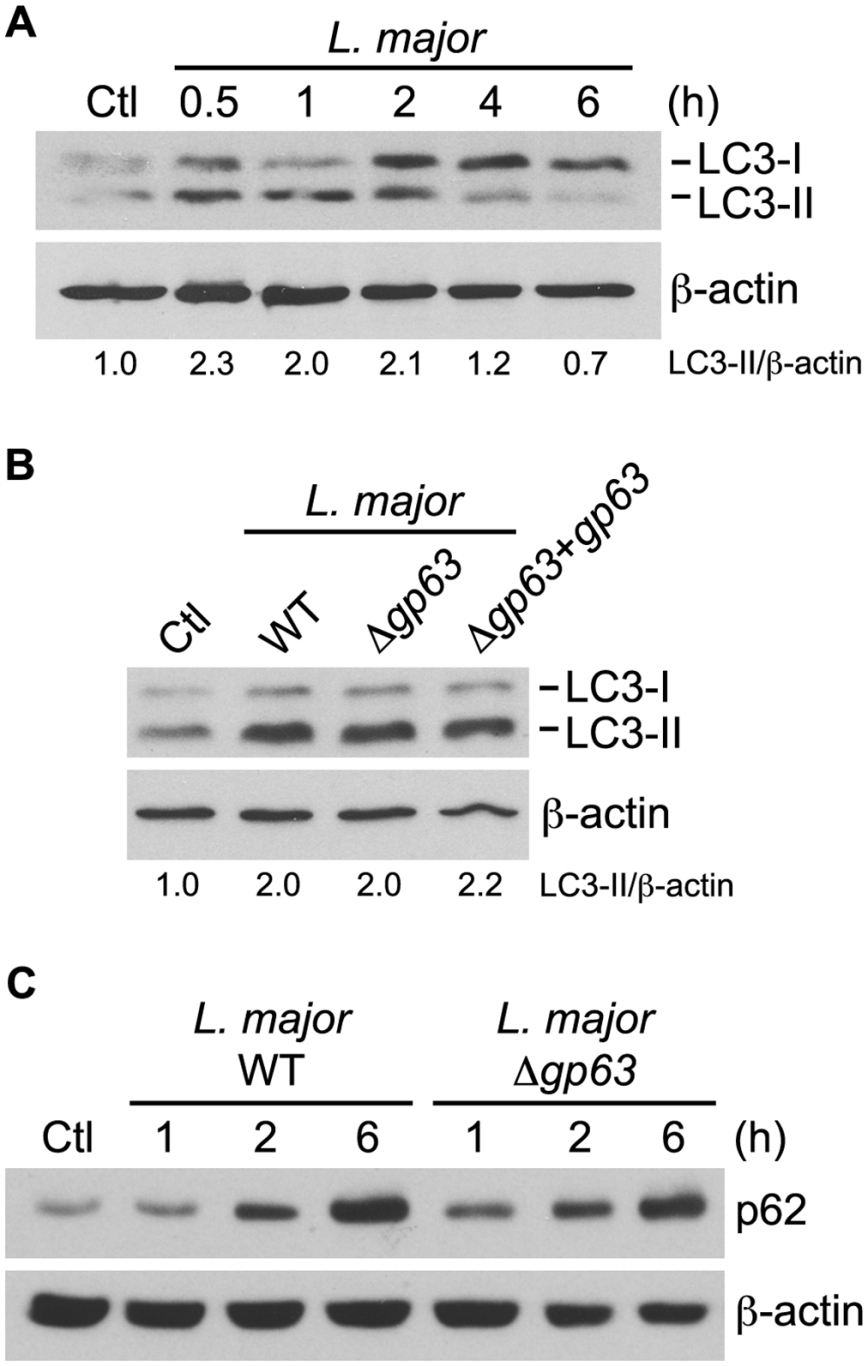
Infection with *L*. *major* promotes LC3 conversion and p62 expression. (A) Kinetics of LC3-I to LC3-II conversion induced by opsonized *L*. *major* NIH S promastigotes in BALB/c BMM was assessed by immunoblot analysis. (B) LC3-I and LC3-II levels in BALB/c BMM infected for 1 h with opsonized WT, Δ*gp63* or Δ*gp63*+gp63 *L*. *major* NIH S promastigotes was assessed by immunoblot analysis. (A, B) LC3-II band intensities were measured by spot densitometry, normalized to the β-actin loading control and compared to the uninfected control (Ctl) cells. (C) Kinetics of p62 accumulation in BALB/c BMM infected with opsonized WT or Δ*gp63 L*. *major* NIH S promastigotes was assessed by immunoblot analysis.

**Fig 2:**
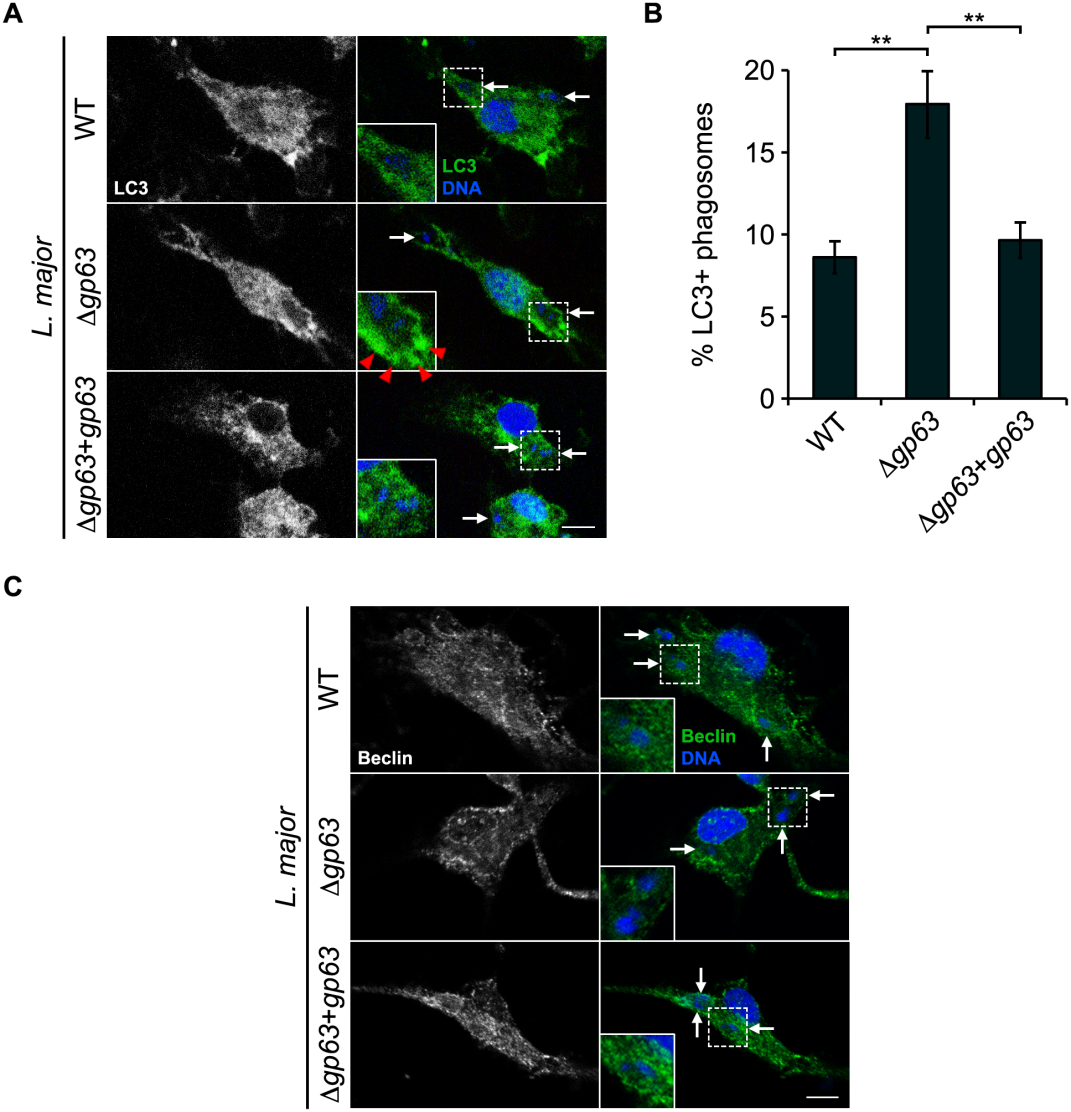
*L*. *major* GP63 prevents LC3 recruitment to phagosomes. (A, C) Confocal microscopy images of BALB/c BMM infected for 1 h with opsonized WT, Δ*gp63*, or Δ*gp63*+*gp63 L*. *major* NIH S promastigotes. LC3 (A) and Beclin (C) are in green; nuclei are in blue. White arrows indicate parasite nuclei; red-filled arrowheads point to LC3 recruitment (A). Scale bar, 5 μM. (B) Quantification of LC3-positive phagosomes at 1 h after infection. Data are presented as the mean ± standard error of the mean (SEM) of values from three independent experiments. **p<0.01.

Growing evidence suggests that LAP contributes to augmenting the microbicidal and immune functions of phagosomes [18, 21–23, 25]. Given the ability of *Leishmania* promastigotes to interfere with phagolysosomal biogenesis, we sought to investigate the impact of *Leishmania* promastigotes on LAP during their internalization by macrophages. Because the GPI-anchored zinc-metalloprotease GP63 interferes with phagolysosomal biogenesis and function [7, 11], it was of interest to determine the potential impact of this molecule on LAP. To this end, we included a *L*. *major* GP63-deficient mutant (Δ*gp63*) and its complemented counterpart (Δ*gp63*+*gp63*) in our study. Recruitment of LC3 to membranes requires the lipidation of cytosolic LC3-I to form membrane-bound LC3-II [27]. We first assessed by Western blot whether internalization of *L*. *major* promastigotes led to the lipidation of LC3 in bone marrow-derived macrophages (BMM). We observed that WT parasites induced a rapid and transient conversion of LC3-I to LC3-II (Fig 1A). Furthermore, GP63-expressing (WT and Δ*gp63*+*gp63*) and Δ*gp63* parasites promoted conversion of LC3-I to LC3-II to a similar extent (Fig 1B), indicating that GP63 is not responsible for this conversion. In addition, expression of the autophagic cargo receptor sequestasome (p62/SQSTM1) increased with time upon infection independently of GP63 (Fig 1C) and was sensitive to cycloheximide. We next determined whether internalization of *L*. *major* promastigotes was accompanied by the recruitment of LC3 to phagosomes. To this end, we incubated macrophages with WT, Δ*gp63*, or Δ*gp63*+*gp63* promastigotes and we examined the intracellular distribution of LC3 by confocal immunofluorescence microscopy. We detected LC3 on less than 10% of phagosomes containing WT promastigotes (Fig 2A and 2B). By contrast, we detected LC3 on a significantly higher percentage (~20%) of phagosomes containing Δ*gp63* parasites (Fig 2A and 2B). As expected, phagosomes containing the Δ*gp63*+*gp63* parasites were similar to WT phagosomes with respect to the presence of LC3. In all cases, the optimal time point for the recruitment of LC3 to phagosomes containing *Leishmania* promastigotes was 1 hour after the initiation of phagocytosis. Recruitment of LC3 to phagosomes was previously shown to be preceded by recruitment of Beclin-1 [18] and was shown to be involved in LAP of *Burkholderia pseudomallei* [28]. In the case of *L*. *major*, we did not detect recruitment of Beclin-1 to phagosomes (Fig 2C). Collectively, these results suggest that *L*. *major* promastigotes interfere with recruitment of LC3 to phagosomes through a GP63-dependent mechanism.

### *Leishmania major* promastigotes block the recruitment of NOX2 to phagosomes in a GP63-dependent manner

**Fig 3:**
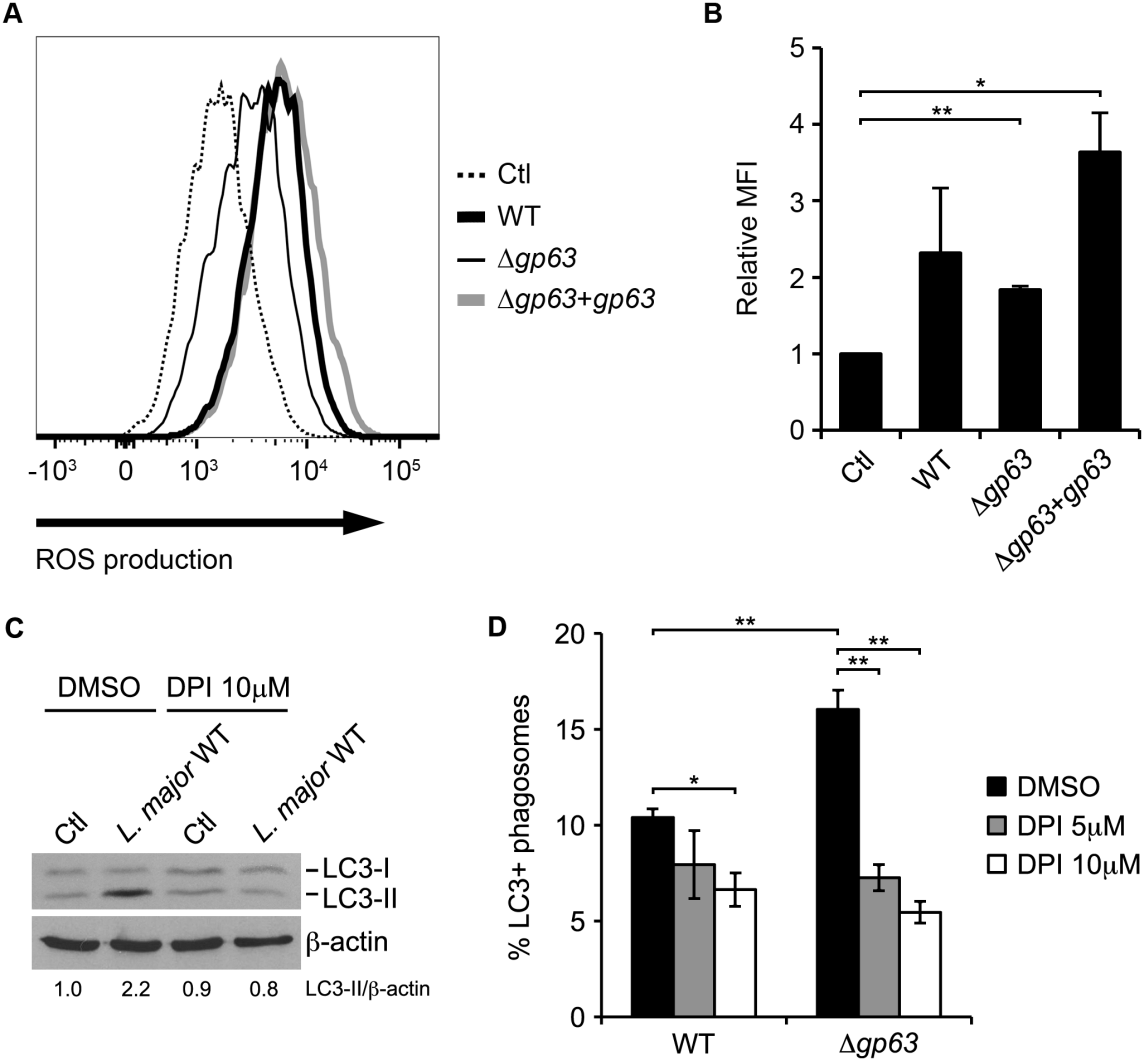
*L*. *major* promastigote-induced ROS production is required for LC3 recruitment to the phagosome. (A, B) Total ROS levels in C57BL/6 x 129 BMM infected for 30 min with opsonized WT, Δ*gp63*, or Δ*gp63*+*gp63 L*. *major* NIH S promastigotes were measured by flow cytometry using CellROX Deep Red Reagent. (A) Representative histograms are shown. (B) Quantification of the mean fluorescence intensity (MFI) of each condition relative to the uninfected control is shown. Data are presented as the mean ± SEM of values from two independent experiments. (C) LC3-I and LC3-II levels in C57BL/6 x 129 BMM infected for 1 h with opsonized WT *L*. *major* NIH S promastigotes, in the presence of 10 μM DPI or of vehicle drug (0.1% DMSO), were assessed by immunoblot analysis. LC3-II band intensities were measured by spot densitometry, normalized to the β-actin loading control and compared to the uninfected control (Ctl) cells cultured in the presence of DMSO. (D) Quantification of LC3-positive phagosomes in BALB/c BMM infected with opsonized *L*. *major* promastigotes NIH S (WT or Δ*gp63*) for 1 h, in the presence of 5 or 10 μM DPI or of vehicle drug (0.1% DMSO). Data are presented as the mean ± SEM of values from triplicate samples. *p<0.05, **p<0.01.

**Fig 4:**
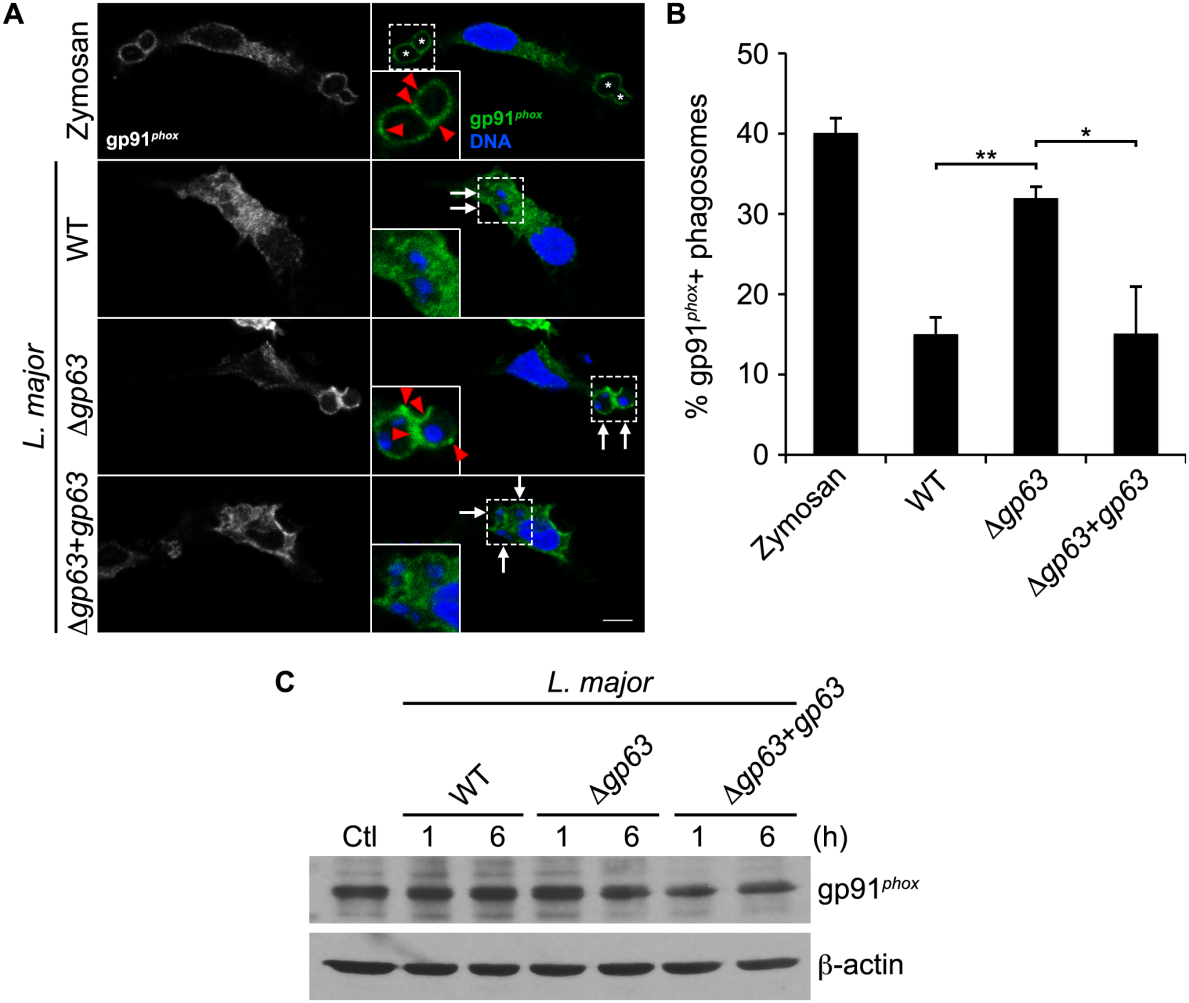
*L*. *major* GP63 inhibits gp91^*phox*^ recruitment to the phagosome. (A) Confocal microscopy images of C57BL/6 peritoneal exudate macrophages (PEM) fed zymosan particles or infected with opsonized WT, Δ*gp63*, or Δ*gp63*+*gp63 L*. *major* NIH S promastigotes for 15 min. gp91^*phox*^ is in green; nuclei are in blue. Asterisks indicate phagosomes containing zymosan particles, white arrows indicate parasite nuclei, and red-filled arrowheads point to gp91^*phox*^ recruitment. Scale bar, 5 μM. (B) Quantification of gp91^*phox*^-positive phagosomes at 15 min after infection. Data are presented as the mean ± SEM of values from three independent experiments. *p<0.05, **p<0.01. (C) gp91^*phox*^ levels in lysates from BALB/c BMM infected with opsonized WT, Δ*gp63* or Δ*gp63*+*gp63 L*. *major* NIH S promastigotes.

During LAP, LC3 is recruited to phagosomes in a NOX2-dependent manner [23, 25]. To elucidate the mechanism by which GP63 contributes to the impairment of this process, we first assessed the ability of WT, Δ*gp63*, and Δ*gp63*+*gp63* promatigotes to activate the production of intracellular ROS in BMM. All three lines activated ROS production, although Δ*gp63* induced lower levels than GP63-expressing parasites (Fig 3A and 3B). We next determined whether conversion of LC3-I to LC3-II induced by *L*. *major* promastigotes was mediated by ROS generated by NOX2. Fig 3C shows that treatment of BMM with the NOX2 inhibitor diphenylene iodonium (DPI) inhibited conversion of LC3-I to LC3-II by WT parasites. Together, these data indicate that *L*. *major* promastigotes activate conversion of LC3-I to LC3-II through the activation of NOX2, independently of the presence of GP63. Importantly, inhibition of NOX2 activity with DPI reduced the recruitment of LC3 to phagosomes containing Δ*gp63* promastigotes to the levels observed for phagosomes containing WT parasites (Fig 3D). These results are consistent with the role of NOX2 in the recruitment of LC3 to phagosomes during LAP [23, 25]. We next determined whether the inhibition of LC3 recruitment to phagosomes containing *Leishmania* promastigotes was associated to a defective phagosomal recruitment of NOX2 (gp91^*phox*^). We infected macrophages with WT, Δ*gp63*, or Δ*gp63*+*gp63* promastigotes and assessed the phagosomal association of gp91^*phox*^ by confocal immunofluorescence microscopy. Zymosan was used as a positive control for the recruitment of gp91^*phox*^. As shown in Fig 4A and 4B, we detected gp91^*phox*^ on nearly 40% of phagosomes containing zymosan and 35% of phagosomes containing Δ*gp63* parasites. In contrast, gp91^*phox*^ was present on approximately 15% of phagosomes containing WT or Δ*gp63*+*gp63* parasites. Given that GP63 cleaves a number of macrophage proteins [29], we next verified whether gp91^*phox*^ was targeted by GP63. Western blot analyses on lysates from macrophages infected with WT, Δ*gp63*, or Δ*gp63*+*gp63* promastigotes showed that gp91^*phox*^ remained intact in infected cells (Fig 4C). Collectively, these results indicate that GP63 enables *L*. *major* promastigotes to prevent recruitment of NOX2 to phagosomes through a mechanism that does not involve its cleavage.

### VAMP8 contributes to the recruitment of LC3 to phagosomes containing *L*. *major*

**Fig 5:**
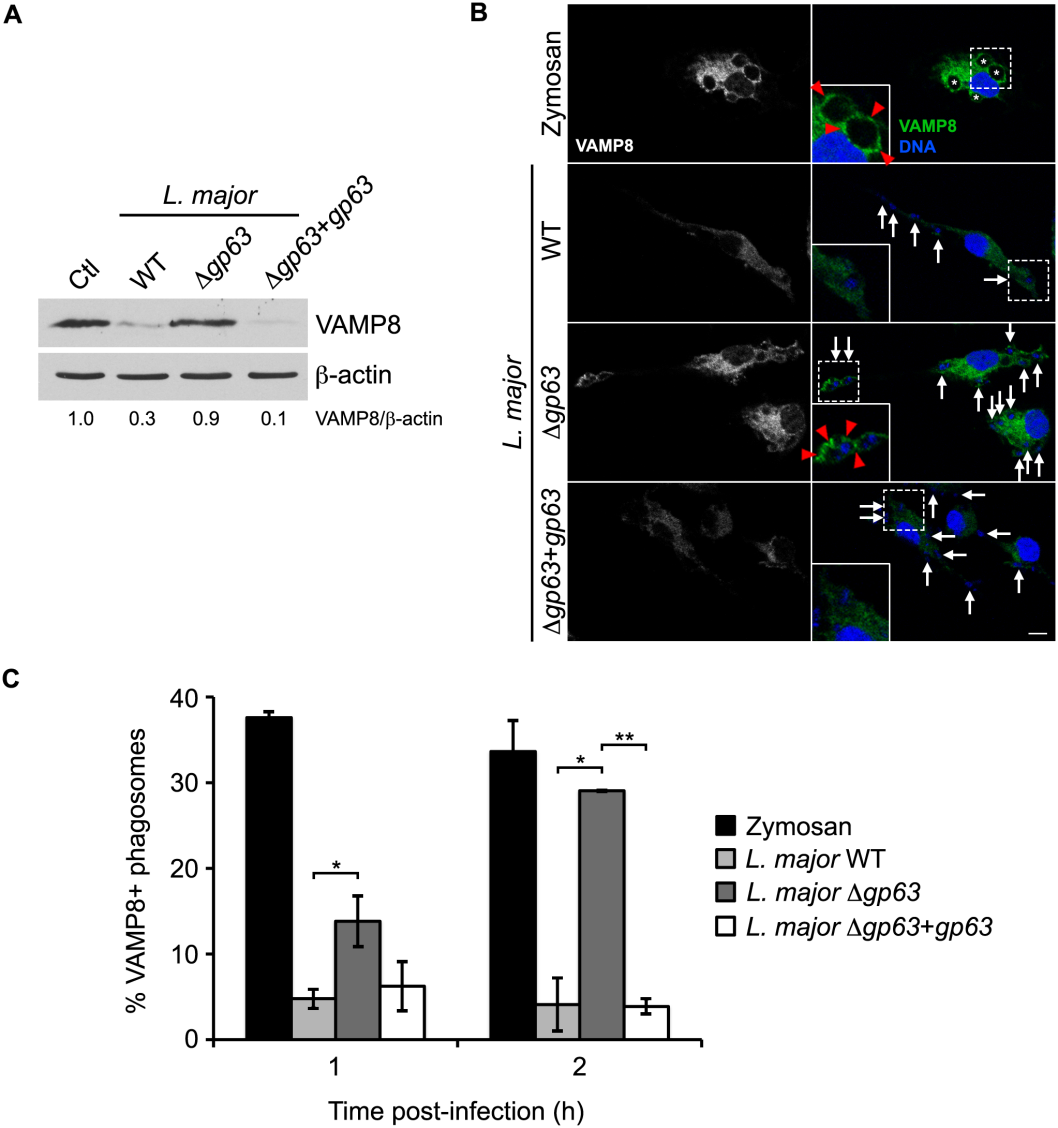
*L*. *major* GP63 cleaves VAMP8 and prevents its recruitment to phagosomes. (A) VAMP8 cleavage in C57BL/6 x 129 BMM infected for 2 h with opsonized WT, Δ*gp63* or Δ*gp63*+*gp63 L*. *major* NIH S promastigotes was assessed by immunoblot analysis. VAMP8 band intensities were measured by spot densitometry, normalized to the β-actin loading control and compared to the uninfected control (Ctl) cells. (B) Confocal microscopy images of C57BL/6 x 129 BMM fed zymosan particles or infected with opsonized WT, Δ*gp63*, or Δ*gp63*+*gp63 L*. *major* NIH S promastigotes for 1 h. VAMP8 is in green; nuclei are in blue. Asterisks indicate phagosomes containing zymosan particles, white arrows indicate parasite nuclei, and red-filled arrowheads point to VAMP8 recruitment. Scale bar, 5 μM. (C) Quantification of VAMP8-positive phagosomes at 1 and 2 h after infection. Data are presented as the mean ± SEM of values from triplicate samples of an experiment representative of more than three others. *p<0.05, **p<0.01.

**Fig 6:**
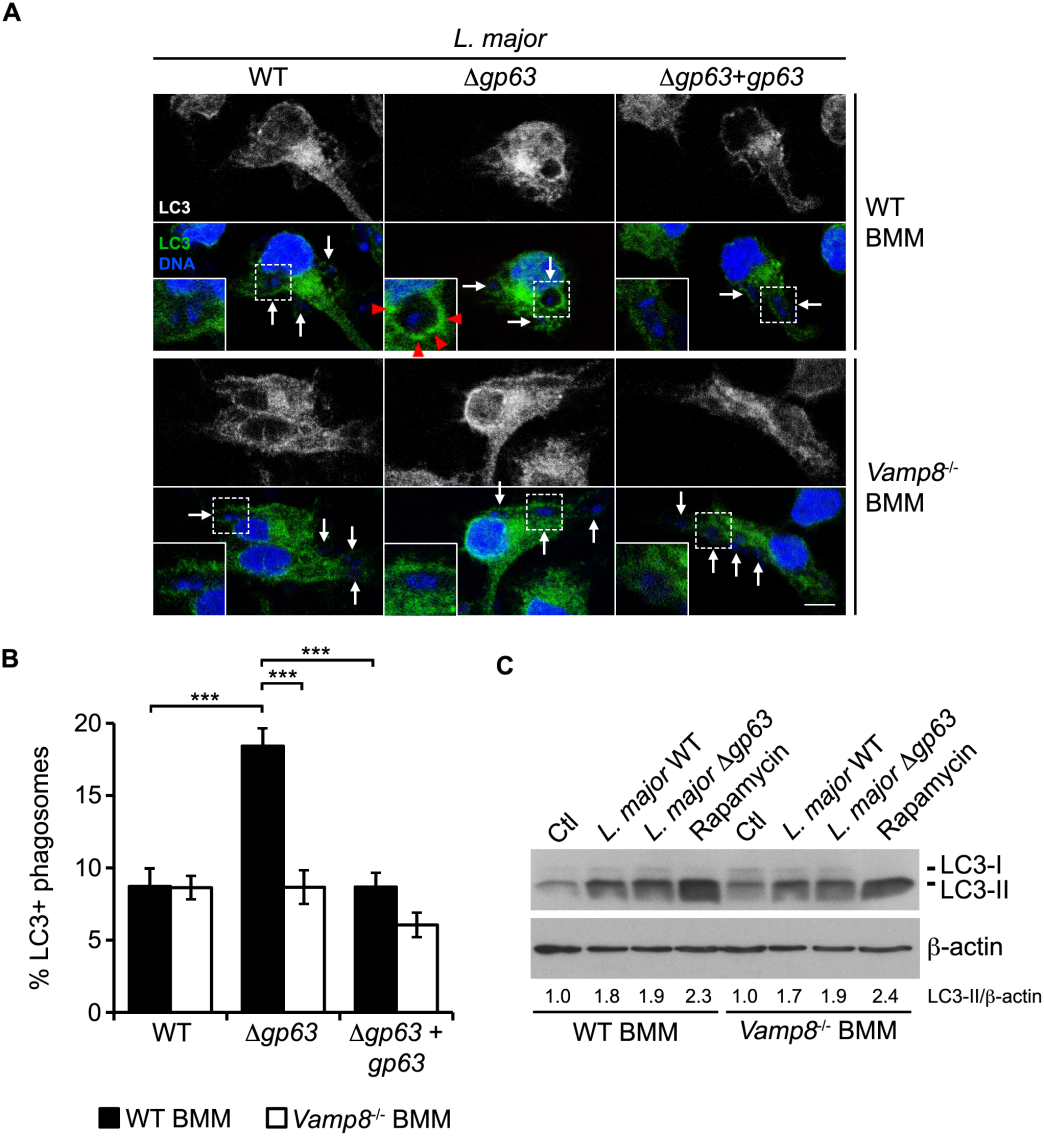
VAMP8 is essential for LC3 recruitment to *L*. *major* parasitophorous vacuoles. (A) Confocal microscopy images of WT or *Vamp8* ^-/-^ C57BL/6 x 129 BMM infected for 1 h with opsonized WT, Δ*gp63*, or Δ*gp63*+*gp63 L*. *major* NIH S promastigotes. VAMP8 is in green; nuclei are in blue. White arrows indicate parasite nuclei, and red-filled arrowheads point to VAMP8 recruitment. Scale bar, 5 μM. (B) Quantification of LC3-positive phagosomes at 1 h after infection. Data are presented as the mean ± SEM of values from three independent experiments. ***p<0.001. (C) LC3-I and LC3-II levels in WT and *Vamp8* ^-/-^ BMM infected for 1 h with opsonized WT or Δ*gp63 L*. *major* NIH S promastigotes or treated with 10 μM rapamycin were assessed by immunoblot analysis. LC3-II band intensities were measured by spot densitometry, normalized to the β-actin loading control and compared to the uninfected, untreated control (Ctl) cells.

We recently showed that VAMP8 regulates the phagosomal recruitment of NOX2 and that *Leishmania* promastigotes cleave and exclude VAMP8 from phagosomes in a GP63-dependent manner [7]. These observations suggested that the GP63-dependent cleavage and exclusion of VAMP8 from phagosomes containing *L*. *major* promastigotes (Figs 5A–5C, S1) may be responsible for the impairment of LAP. To test this hypothesis, we used BMM from VAMP8 null mice. We first assessed the impact of VAMP8 on the recruitment of LC3 to phagosomes. In WT BMM, we observed a two-fold decrease in the recruitment of LC3 to phagosomes containing WT or Δ*gp63*+*gp63* promastigotes compared to phagosomes containing Δ*gp63* parasites (Fig 6A and 6B). In contrast, in *Vamp8* ^*-/-*^ BMM, the recruitment of LC3 to phagosomes containing Δ*gp63* was similar to that observed for phagosomes containing either WT or Δ*gp63*+*gp63* promastigotes and was comparable to the levels observed in WT BMM infected with WT parasites (Fig 6A and 6B). We ensured that VAMP8 was not required for *L*. *major*- or rapamycin-induced conversion of LC3-I to LC3-II (Fig 6C). The non-pathogenic *L*. *tarentolae* species, which does not express a functional GP63 [30] and does not cleave VAMP8 (7), induced LC3 recruitment to the same extent as *L*. *major* Δ*gp63*, further corroborating these results (S2 Fig). Altogether, these results indicate that VAMP8 plays a role in LAP and suggest that *L*. *major* interferes with LAP through GP63-mediated targeting of VAMP8.

### Absence of VAMP8 does not affect intracellular survival of *L*. *major* in macrophages

**Fig 7:**
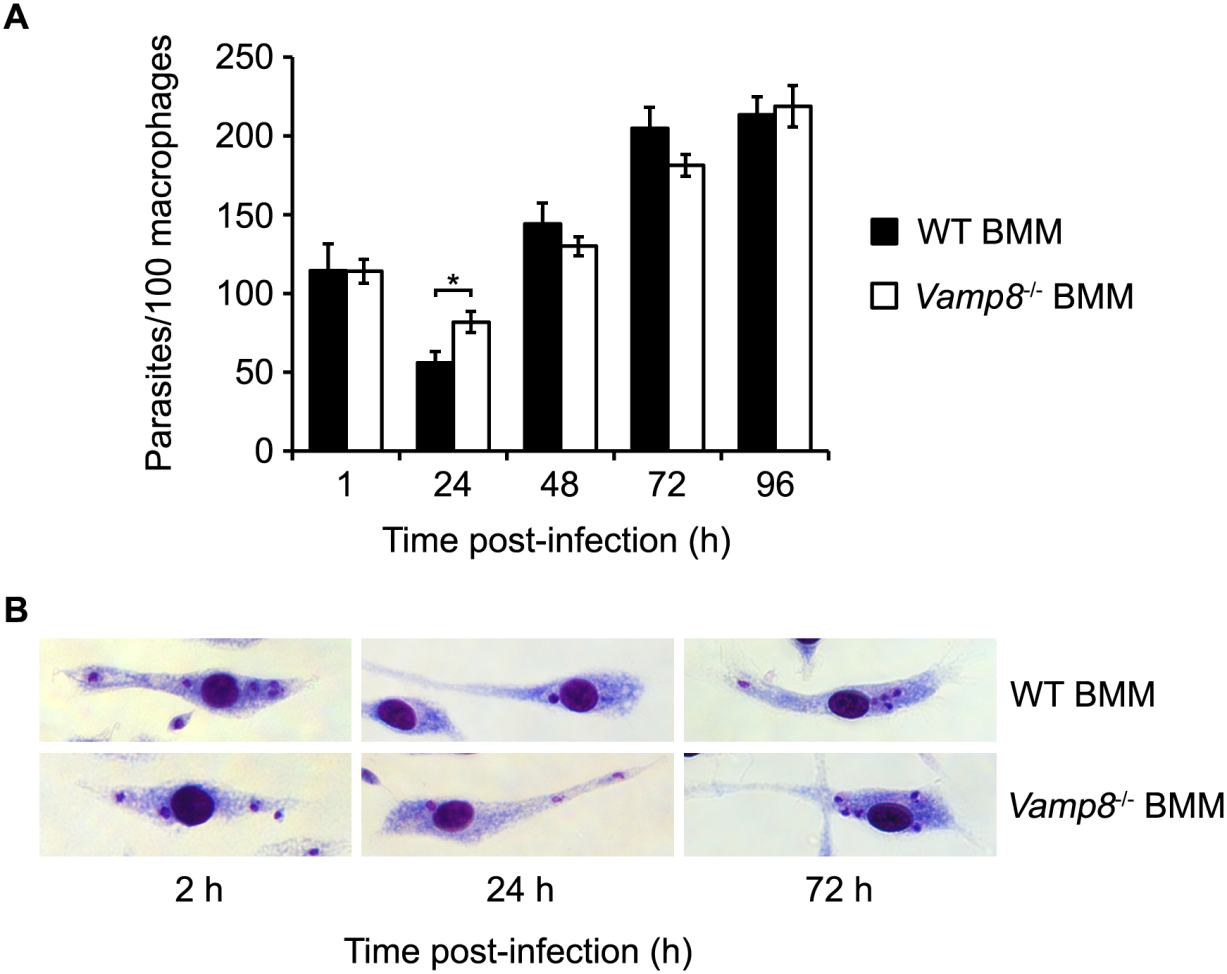
Absence of VAMP8 does not affect intracellular survival of *L*. *major* in macrophages. (A) Quantification of opsonized *L*. *major* GLC94 promastigotes internalization and replication in WT or *Vamp8* ^-/-^ C57BL/6 x 129 BMM, assessed by Giemsa staining at 1, 24, 48, 72, and 96 h after infection. Data are presented as the mean ± SEM of values from three independent experiments. *p<0.05. (B) Images of Giemsa-stained WT or *Vamp8* ^-/-^ BMM infected with opsonized *L*. *major* GLC94 for 2, 24, and 72h.

LAP was shown to restrict or contribute to the establishment of pathogens such as *Salmonella* Typhimurium, *Burkholderia pseudomallei*, *Aspergillus fumigatus*, and *Listeria monocytogenes* within their host cells [23, 31–33]. Given the involvement of VAMP8 in LAP, we evaluated the impact of VAMP8 on the control of *Leishmania* infection in macrophages. To this end, we infected WT and *Vamp8* ^*-/-*^ BMM with *L*. *major* promastigotes and assessed parasite survival and replication over 96 h. As shown in Fig 7A, *L*. *major* promastigotes were internalized to the same extent by WT and *Vamp8* ^*-/-*^ BMM. Furthermore, we observed no differences in the survival and replication of *L*. *major* promastigotes in the absence of VAMP8 over 96 h, with the exception of a small but significant increase in parasite survival in *Vamp8* ^*-/-*^ BMM at 24 h post-infection (Fig 7A). Moreover, we observed no obvious differences in parasitophorous vacuole size and appearance in the absence of VAMP8 (Fig 7B). These results are consistent with the ability of *L*. *major* to efficiently neutralize VAMP8 and prevent its recruitment to phagosomes through the action of GP63.

## Discussion

Establishment of an intracellular infection by *Leishmania* promastigotes is characterized by an inhibition of phagosome maturation and function [7, 8, 34, 35]. Whilst phagosome remodeling is mostly achieved through LPG-mediated disruption of lipid rafts [10, 36], recent evidence indicates that the *Leishmania* metalloprotease GP63 contributes to this process through cleavage of membrane fusion regulators [7, 11, 15]. In the present study, we provide evidence that *L*. *major* promastigotes evade LC3-associated phagocytosis, a non-canonical autophagic process normally involved in the elimination of pathogens through the enhancement of phagosome maturation [18, 23]. We showed that recruitment of the autophagy protein LC3 to phagosomes containing *L*. *major* promastigotes is impaired through GP63-mediated cleavage of VAMP8. These findings are consistent with the notion that targeting components of the host cell membrane fusion machinery contributes to the ability of *Leishmania* promastigotes to alter phagosomal properties and modulate host immune responses during the establishment of infection [7, 15].

ROS play a central role in the biology of phagocytes [32]. In addition to their key roles in microbial killing and antigen cross-presentation, ROS generated by NOX2 are necessary for LAP [23, 25]. Recruitment and activation of the NOX2 complex to phagosomes during LAP is a highly regulated process that is starting to be understood. The protein Rubicon was recently shown to be required for the recruitment of LC3 to phagosomes [33]. This protein facilitates sustained levels of phosphatidylinositol-3 phosphate on phagosomes and stabilizes the NOX2 complex for production of ROS [33]. The FYCO1 protein, which is involved in autophagosome trafficking, was shown to limit ROS production by phagosomes during Dectin-1-mediated phagocytosis and to facilitate the maturation of early p40^*phox*^-positive phagosomes into LAMP1-positive phagosomes [37]. Here, we identified VAMP8 as a regulator of LC3 recruitment to phagosomes, consistent with the role of this SNARE in the phagosomal recruitment of NOX2 [7]. We found that activation of ROS production by *L*. *major* promastigotes is required for LC3 lipidation and, consistent with previous studies, we also found that inhibition of NOX2 impaired the recruitment of LC3 to phagosomes harboring *L*. *major*. Furthermore, *L*. *major* promastigotes efficiently excluded NOX2 from formed phagosomes in a GP63-dependent mechanism. However, this GP63-mediated exclusion of NOX2 from phagosomes did not involve its proteolytic cleavage. Rather, through the action of GP63 on VAMP8, the NOX2 complex is not assembled on phagosomes, precluding LC3 recruitment. It is interesting to note that the intracellular bacterium *Legionella* interferes with host autophagy by directly targeting LC3 through the action of the effector protein RavZ, a cysteine protease injected into the host cell cytosol via a type IV secretion system [38]. In the case of GP63, determining how this protease exits phagosomes is an important issue to resolve in order to understand how it cleaves its substrates, including VAMP8, within infected cells. Indeed, despite the absence of known secretion system in *Leishmania*, GP63 redistributes within infected cells in vesicular structures throughout the cytoplasm [7, 15], where it may come in close proximity of VAMP8 present on late endosomes and lysosomes. This issue is currently under investigation.

The possible consequences of preventing phagosomal LC3 recruitment may be inferred from a recent study aimed at investigating the role of LC3 during Dectin-1-mediated phagocytosis in bone marrow-derived dendritic cells [25]. Hence, Dectin-1-triggered phagocytosis, ROS, microbial killing, and cytokine production in response to β-glucan particles were normal in the absence of LC3. On the other hand, absence of LC3 reduced the ability of dendritic cells to present antigen in the context of MHCII and to activate CD4 T cells. This was the consequence of a reduced ability of phagosomes to acquire MHCII molecules in the absence of LC3. Interestingly, the defect in antigen presentation observed in LC3-deficient dendritic cells is specific to MHC class II [25]. How LC3 influences MHC class II recruitment to maturing phagosomes is not yet known. Given that *Leishmania* alters the antigen-presenting and immunomodulatory functions of dendritic cells through multiple mechanisms [7, 39, 40], it may be complicated to assess to which extent the exclusion of LC3 from *L*. *major*-harboring PV contributes to the ability of these parasites to evade MHC class II-dependent antigen presentation. Indeed, we recently showed that the *L*. *major* inhibits MHC class II antigen presentation in a GP63-dependent manner. This inhibition was not due to the cleavage of VAMP8 by GP63, since MHC class II presentation was not altered in *Vamp8* ^*-/-*^ cells [7]. Of note, Crauwels and colleagues [41] investigated the fate of apoptotic *Leishmania* promastigotes in human macrophages. They observed that in contrast to viable parasites, the majority of apoptotic promastigotes were internalized in LC3-positive phagosomes. In our study, we detected less than 1% apoptotic parasites in the stationary phase promastigote populations we used to infect macrophages. Thus, in our experimental system, the absence of GP63 rather than a difference in the proportion of apoptotic promastigotes among WT, Δ*gp63*, or Δ*gp63*+*gp63* parasites cultures is responsible for the increased proportion of LC3-positive phagosomes observed during the phagocytosis of *L*. *major* Δ*gp63* parasites. Whether the apoptotic *L*. *major* parasites populations used by Crauwels *et al*. contain less GP63 than the viable parasites is not known.

The contribution of VAMP8 in LAP is consistent with its role in the recruitment of NOX2 to phagosomes [7]. However, our data do not exclude the possibility that VAMP8 could also be involved in the fusion between LC3-positive autophagosomes and phagosomes. Indeed, a role for VAMP8 in xenophagy has been described during the invasion of HeLa cells by group A *Streptococcus* (GAS) [42]. In that study, VAMP8 was shown to act in concert with another SNARE, Vti1b, to mediate the fusion of xenophagosomes with lysosomes and the recruitment of LC3 to the resulting compartments. Consistently, the bactericidal efficiency of HeLa cells was reduced in the absence of VAMP8 [42]. In the case of *Leishmania* promastigotes, we found that the absence of VAMP8 had no significant impact on the survival and replication of the parasite inside macrophages. Thus, as shown for *Candida albicans* [25], LAP may not play a significant role during the establishment of *L*. *major* infection within macrophages. This may be related to the fact that *Leishmania* promastigotes can efficiently target VAMP8 during the infection process.

In sum, we discovered that *L*. *major* promastigotes evade LAP through the action of a metalloprotease, GP63, by excluding NOX2 from phagosomes, thereby highlighting a novel strategy exploited by an intracellular pathogen to interfere with the host antimicrobial machinery.

## Materials and Methods

### Ethic statements

All animals were handled in strict accordance with good animal practice as defined by the Canadian Council on Animal Care, and all animal work was approved by the Comité Institutionnel de Protection des Animaux of the Institut National de la Recherche Scientifique-Institut Armand-Frappier (protocol 1302–03). This protocol respects procedures on good animal practice provided by the Canadian Council on Animal Care (CCAC).

### Macrophage culture

BMM were obtained from the femurs and tibias of 6- to 8-week-old female BALB/c (Charles River), C57BL/6 or *Vamp8* ^*-/-*^ mice and their wild type (WT) counterpart (on a mixed C57BL/6 x 129 genetic background) described elsewhere [43] and differentiated as previously described [44] in complete medium (Dulbecco’s Modified Eagle’s Medium with glutamine (Thermo Fisher Scientific), containing 10% heat-inactivated, fetal bovine serum (FBS) (PAA laboratories Inc.), 10 mM HEPES pH 7.4 and penicillin-streptomycin), supplemented with 15% (v/v) L929 cell-conditioned medium as a source of colony-stimulating factor-1 (CSF-1), in a 37°C incubator with 5% CO_2_. BMM were made quiescent by culturing them in the absence of CSF-1 for 18 h prior to infection. Where indicated, BMM were treated for the indicated times with 10 μM rapamycin (Cayman Chemical Co.) as a positive control for autophagy induction or with 5 to 10 μM of the NADPH oxidase inhibitor DPI (Sigma-Aldrich). PEM were obtained from female C57BL/6 mice by peritoneal lavage 3 days after peritoneal injection of 3% (w/v) proteose-peptone and were maintained in complete medium. These macrophages were used to assess gp91^*phox*^ recruitment to phagosomes, as the detection of this molecule by confocal immunofluorescence microscopy was superior than in BMM.

### Parasites and infections

Promastigotes of *L*. *major* NIH S (MHOM/SN/74/Seidman) clone A2 and *L*. *major* GLC94 (MHOM/TN/95/GLC94 zymodeme MON25) were cultured at 26°C in *Leishmania* medium (M199 medium supplemented with 10% heat-inactivated FBS, 100 μM hypoxanthine, 10 mM HEPES, 5 μM hemin, 3 μM biopterin, 1 μM biotin, and penicillin-streptomycin). The *L*. *major* NIH clone A2 isogenic Δ*gp63* mutant and its complemented counterpart Δ*gp63*+*gp63* have been previously described [45]. Cultures of Δ*gp63*+*gp63* promastigotes were supplemented with 50 μg/ml G418. For BMM infections, promastigotes were used at the late stationary phase of growth. Complement opsonization of promastigotes and zymosan particles was performed prior to phagocytosis by incubating the particles in phosphate-buffered saline (PBS) containing 10% mouse serum for 30 min at 37°C. BMM were then incubated at 37°C with promastigotes (parasite-to-macrophage ratio of 15:1) or zymosan particles (ratio of 5:1) for the indicated times. Infection levels were assessed by microscopic examination of infected cells after Giemsa staining with the Hema 3 system (Fisher Scientific).

### Western blotting

Adherent BMM were washed with ice-cold PBS containing 1 mM Na_3_VO_4_ and lysed in 50 mM Tris-HCl pH 8, 150 mM NaCl and 1% Nonidet P-40, containing complete protease inhibitors (Roche Applied Science) and phosphatase inhibitors (1 mM Na_3_VO_4_, 50 mM NaF, 1.5 mM EGTA and 10 mM Na_4_P_2_O_7_). When assessing GP63-mediated cleavage of host proteins, the zinc chelator 1,10-phenanthroline (Sigma-Aldrich) [46] was added to the lysis buffer, at a concentration of 10 mM, to ensure that the observed proteolytic cleavage occurs during the infection process rather than during sample preparation. Insoluble material was removed by centrifugation for 10 min at 4°C and protein concentrations were determined using the Pierce BCA protein assay kit (Pierce). For LC3 conversion analysis, cells were lysed in RIPA buffer (50 mM Tris-HCl pH 8.0, 150 mM NaCl, 0.1% SDS, 0.5% Na deoxycholate and 1% NP-40) containing protease and phosphatase inhibitors and whole cell extracts were briefly sonicated before insoluble material was removed by centrifugation for 10 min at 4°C. Samples were separated by SDS-PAGE on 6 to 15% polyacrylamide gels and then transferred to Hybond-LFP PVDF membranes (LC3) or Hybond-ECL nitrocellulose membranes (GE Healthcare Life Sciences) using a Trans-Blot SD Semi-Dry Transfer Cell apparatus (BioRad). Membranes were blocked with 5% BSA and incubated with the relevant antibodies. For immunodetection, horseradish peroxidase (HRP)-conjugated anti-mouse or anti-rabbit IgG and the enhanced chemiluminescence (ECL) detection reagents from GE Healthcare Life Sciences were used. The rabbit polyclonal antibody against LC3B was purchased from Novus Biologicals. Rabbit polyclonal antiserum against VAMP8 was from Synaptic Systems. Mouse monoclonal antibodies against p62 (2C11), gp91^*phox*^ (clone 53), and β-actin (A5316) were purchased from Abnova Corporation, BD Transduction Laboratories, and Sigma-Aldrich, respectively. When indicated, radiographic films were scanned with the AlphaImager 3400 imaging system (Alpha Innotech Corporation), which is equipped with a 12-bit CCD camera and generates high-resolution, 16-bit files. All images were free of pixel saturation. Band intensities in each condition were quantified by spot densitometry, normalized to their respective β-actin loading control and compared to the uninfected, untreated control.

### Confocal immunofluorescence microscopy

BMM were seeded in 24-well plates containing microscope coverslips (Fisher Scientific) and infected with *L*. *major* promastigotes or fed zymosan particles for the indicated times. Assessment of the phagosomal recruitment of gp91^*phox*^ was performed with PEM, as the signal was superior to the signal obtained with BMM. Cells were washed with PBS, fixed with 2% paraformaldehyde (PFA) for 10 min and then simultaneously blocked and permeabilized in 0.1% Triton X-100, 1% bovine serum albumin, 20% normal goat serum, 6% non-fat dry milk and 50% FBS for 20 min. Cells were incubated for 1h to 2h with antibodies against LC3B (1:200), Beclin-1 (rabbit polyclonal, Abcam; 1:300), gp91^*phox*^ (1:70), or VAMP8 (1:100). AlexaFluor 488-conjugated goat anti-rabbit IgG or goat anti-mouse IgG (1:500, Thermo Fisher Scientific) was used for 30 min, during which time macrophage and promastigote nuclei were stained with DRAQ5 (1:400, BioStatus Ltd.). Coverslips were washed three times with PBS between incubations and all steps were performed at room temperature. Coverslips were then mounted on Fluoromount-G (SouthernBiotech) and sealed with nail polish. Analyses of LC3 distribution were performed with a Bio-Rad Radiance 2000 confocal imaging system (Bio-Rad Laboratories) installed on an Eclipse E800 microscope, using an argon/krypton laser at 488 nm with a Plan Apo Nikon 60X (NA 1.4) oil immersion lens for LC3 fluorescence and a 638 nm diode laser at 650 nm long-pass with a Plan Apo Nikon 60x (NA 1.4) oil immersion lens for DRAQ5 fluorescence. Images were acquired in the normal scanning mode with a Kalman filter of 3 to 6 using the LaserSharp software. Analyses for Beclin, gp91^*phox*^ and VAMP8 were performed with a Plan APOCHROMAT 63x oil-immersion DIC 1.4NA objective on a Zeiss LSM780 confocal microscope (Carl Zeiss Microimaging) equipped with 30 mW 405 nm diode laser, 25mW 458/488/514 argon multiline laser, 20mW DPSS 561 nm laser and 5mW HeNe 633 nm laser and mounted on a Zeiss Axio Observer Z1. Images were acquired in plane scanning mode and were minimally and equally processed using Carl Zeiss ZEN 2012 software. For all experiments, a minimum of 100 phagosomes per coverslip were examined for every experimental condition, each performed in duplicates or triplicates, as indicated. Experiments were repeated the indicated number of times prior to statistical analysis.

### ROS measurement

Adherent BMM were infected with *L*. *major* NIH S promastigotes for 30 min at 37°C in the presence of 5 μM CellROX Deep Red Reagent (Thermo Fisher Scientific). Subsequently, after two washes with PBS, cells were collected by scraping in PBS, fixed for 10 min in 4% PFA and washed twice with PBS. Samples were acquired in the APC channel on a LSRFortessa SORP cytometer with the BD FACSDiva6.2 software (BD Biosciences). Fluorescence intensity values were analyzed using the FlowJo software (version 10; TreeStar).

### Statistical analysis

The two-tailed, unpaired Student’s t test was performed to evaluate the significance of the differences observed. * p < 0.05, ** p < 0.01, *** p < 0.001.

## Supporting Information

S1 Fig *L*. *major* GP63 down-modulates VAMP8. Confocal microscopy images of C57BL/6 x 129 BMM from wild type or *Vamp8* ^-/-^ mice infected for 1 h with opsonized WT, Δ*gp63*, or Δ*gp63*+*gp63 L*. *major* promastigotes. VAMP8 is in green; nuclei are in blue. Fields containing several cells are shown to display the decrease in VAMP8 intensity upon infection with GP63-expressing parasites. Asterisks indicate phagosomes containing zymosan particles and white arrowheads point to parasite nuclei. Scale bar, 5 μM.
(JPG)

S2 Fig *L*. *tarentolae* does not prevent LC3 recruitment to the phagosome. (A) Confocal microscopy images of BALB/c BMM infected for 1 h with opsonized *L*. *major* or *L*. *tarentolae* promastigotes. LC3 is in green; nuclei are in blue. White arrows indicate parasite nuclei; red-filled arrowheads point to LC3 recruitment. Scale bar, 5 μM. (B) Quantification of LC3-positive phagosomes at 1 h after infection. Data are presented as the mean ± SEM of values from two independent experiments. ***p<0.001.
(JPG)
