# Supplementary figures and images for: Leishmania major Promastigotes Evade LC3-Associated Phagocytosis through the Action of GP63
Source: PLoS Pathog. 2016 Jun 9;12(6):e1005690. doi: 10.1371/journal.ppat.1005690 (PMC4900527; doi:10.1371/journal.ppat.1005690)

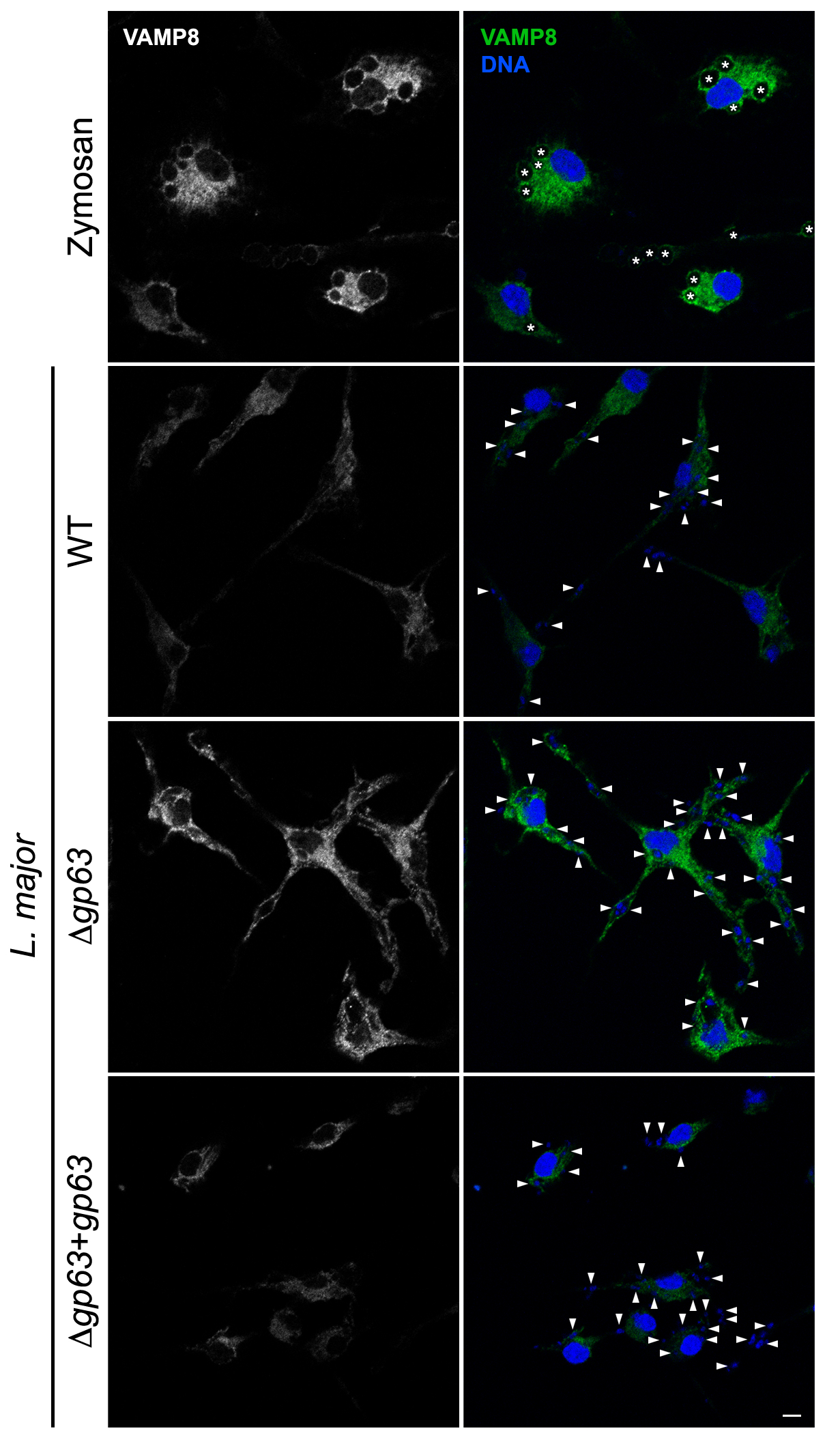

Supplement: S1 Fig — Confocal microscopy images of C57BL/6 x 129 BMM from wild type or Vamp8 -/- mice infected for 1 h with opsonized WT, Δgp63, or Δgp63+gp63 L. major promastigotes. VAMP8 is in green; nuclei are in blue. Fields containing several cells are shown to display the decrease in VAMP8 intensity upon infection with GP63-expressing parasites. Asterisks indicate phagosomes containing zymosan particles and white arrowheads point to parasite nuclei. Scale bar, 5 μM. (JPG) [file ppat.1005690.s001.jpg]

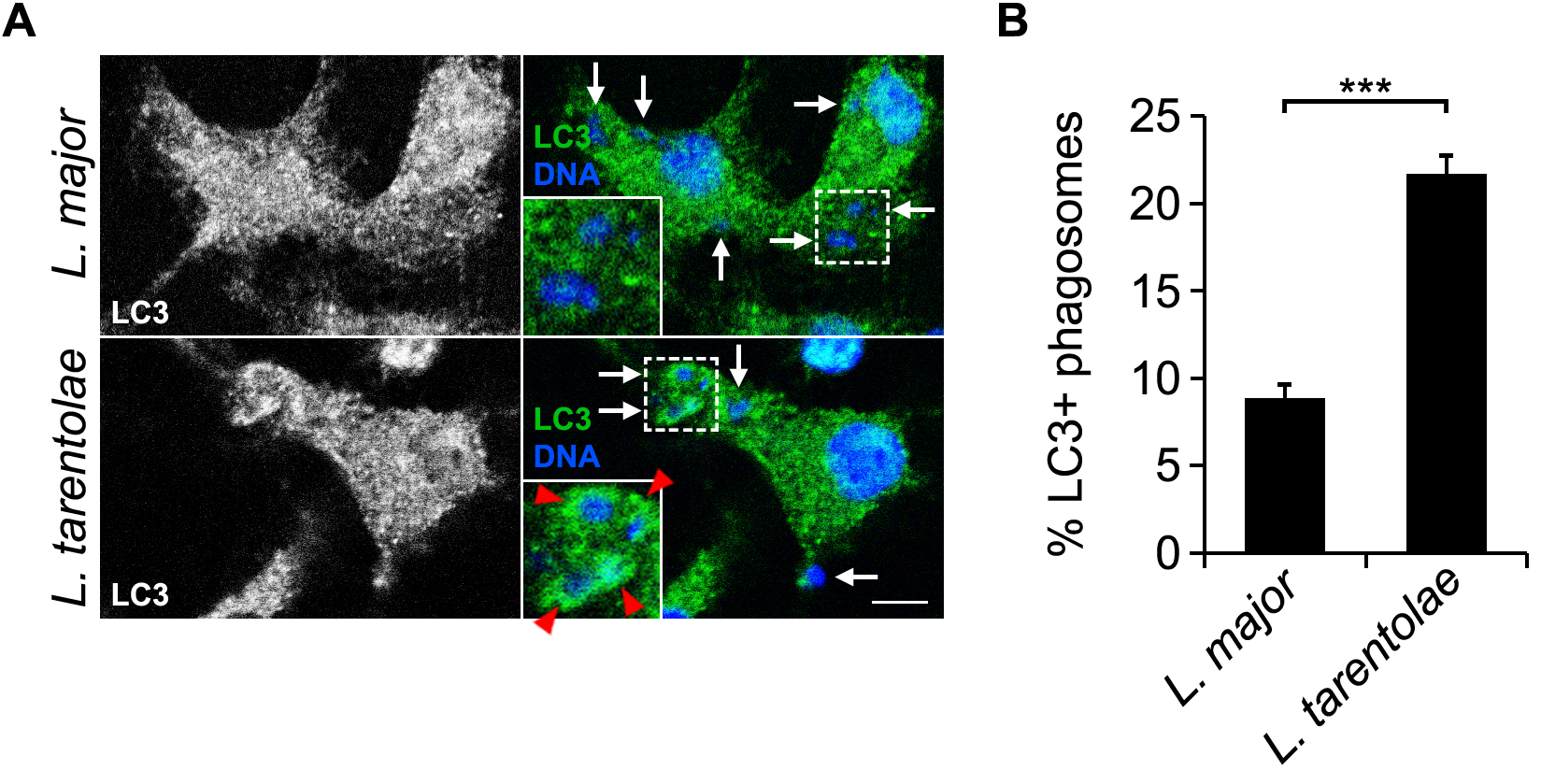

Supplement: S2 Fig — (A) Confocal microscopy images of BALB/c BMM infected for 1 h with opsonized L. major or L. tarentolae promastigotes. LC3 is in green; nuclei are in blue. White arrows indicate parasite nuclei; red-filled arrowheads point to LC3 recruitment. Scale bar, 5 μM. (B) Quantification of LC3-positive phagosomes at 1 h after infection. Data are presented as the mean ± SEM of values from two independent experiments. ***p<0.001. (JPG) [file ppat.1005690.s002.jpg]
